# Penicillin skin testing in methicillin-sensitive staphylococcus aureus bacteremia: A cost-effectiveness analysis

**DOI:** 10.1371/journal.pone.0210271

**Published:** 2019-01-07

**Authors:** T. Joseph Mattingly, Stephen Meninger, Emily L. Heil

**Affiliations:** University of Maryland School of Pharmacy, Baltimore, Maryland, United States of America; Albert-Ludwigs-Universitat Freiburg, GERMANY

## Abstract

**Background:**

Beta-lactams are the mainstay for treating methicillin-susceptible *Staphylococcus aureus* (MSSA) infections complicated by bacteremia due to superior outcomes compared with vancomycin. With approximately 11% of inpatients reporting a penicillin (PCN) allergy, many patients receive suboptimal treatment for MSSA bacteremia.

**Objective:**

Evaluate the cost-effectiveness of penicillin skin testing (PST) in adult patients with self-reported PCN allergy in an inpatient setting undergoing treatment for MSSA bacteremia.

**Methods:**

A decision analytic model was developed comparing an acute care PST intervention to a scenario with no confirmatory allergy testing. The primary outcome was the incremental cost-effectiveness ratio (ICER) from the health-sector perspective over a 1-year time horizon using quality-adjusted life years (QALYs) as the measure for effectiveness. One-way and probabilistic sensitivity analyses were conducted to assess the uncertainty of the ICER estimation.

**Results:**

Over a 1-year time horizon, PST services applied to all MSSA bacteremia patients reporting a PCN-allergy would result in a cost per patient of $12,559 and 0.73 QALYs while no PST services would have a higher cost per patient of $13,219 and 0.66 QALYs per patient. This resulted in a cost-effectiveness estimate of -$9,429 per QALY gained. Varying the cost of implementing PST services determined a break-even point of $959.98 where any PST cost less than this amount would actually be cost saving.

**Conclusions:**

Patients reporting a PCN allergy on admission may receive sub-optimal alternative therapies to beta-lactams, such as vancomycin, for MSSA bacteremia. This economic analysis demonstrates that inpatient PST services confirming PCN allergy are cost-effective for patients with MSSA bacteremia.

## Introduction

*Staphylococcus aureus* is a leading cause of bacteremia that is associated with high mortality rates and represents a significant burden to the healthcare system.[1, 2] Beta-lactams are the mainstay for treating methicillin-susceptible *Staphylococcus aureus* (MSSA) infections complicated by bacteremia due to superior outcomes compared with vancomycin.[3–7] However, approximately 11% of inpatients report a penicillin (PCN) allergy, limiting optimal treatment for MSSA bacteremia.[8]

Penicillin skin testing (PST) assesses local reactions to the major and minor determinants of type I reactions with a negative predictive value of 97–99%.[9, 10] It is estimated only 1% of the general population is truly allergic to penicillin and that less than 10% of patients with penicillin allergy histories who received PST are found to be at risk for an acute allergic reaction.[11] Therefore ruling out penicillin allergy through PST would allow for antibiotic optimization in the treatment of many infectious conditions including MSSA bacteremia. Unfortunately, availability of PST in an inpatient setting is limited in many facilities due to lack of time or personnel.[12, 13] The objective of this study was to estimate the cost-effectiveness of PST in adult patients in an inpatient setting undergoing treatment for MSSA bacteremia.

## Methods

### Model structure

**Fig 1:**
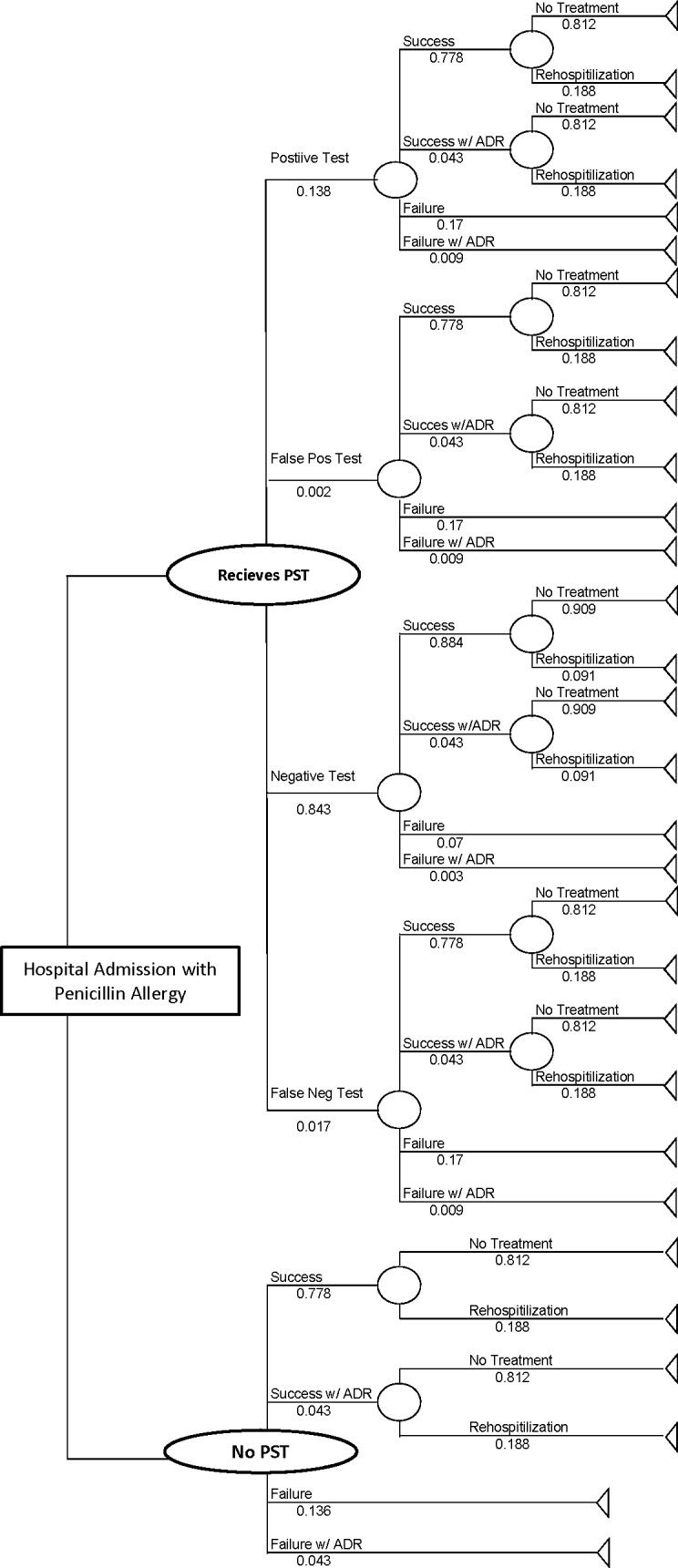
Decision-analytic model for penicillin skin testing (PST) in bacteremia.

We developed a decision analytic model using Microsoft Excel (Santa Rosa, California) to evaluate the cost-effectiveness of an acute care penicillin skin testing (PST) intervention for all patients admitted with MSSA bacteremia who self-report an allergy to PCN compared with the standard of care scenario with no confirmatory allergy testing. Once a treatment decision was made, path possibilities included treatment success with no ADE, treatment success with ADE, treatment failure with no ADE, and treatment failure with ADE. Patients receiving PST had four additional branch scenarios: true positive, true negative, false positive, false negative (Fig 1).

The primary outcome was the incremental cost-effectiveness ratio (ICER) from the health-sector perspective over a 1-year time horizon using quality-adjusted life years (QALYs) as the primary outcome measure for effectiveness. Limited evidence for the indirect cost or health benefits prevented the ability to determine a value from the societal perspective. This study was not considered human research according to the authors’ Institutional Review Board.

### Model parameters

All model inputs for the base case scenario along with parameter distribution assumptions are presented in Table 1. Clinical effectiveness and mortality inputs focused on the probability of antibiotic treatment success for MSSA bacteremia in patients admitted with a reported PCN allergy with no confirmatory testing and with PST-guided therapy.[14] The PST-guided therapy assumptions in the acute setting were based on treatment with cefazolin versus vancomycin as described by Blumenthal et al.[14] Outpatient antibiotic estimates were based off of outpatient studies by Sade et al. and Macy.[15, 16] Patients in PST-guided therapy branches included probabilities of skin test errors based on previously reported sensitivity and specificity of the procedure.[17] For patients with no confirmatory PST testing, it was assumed that “self-reported” allergies guided the treatment decision between cefazolin and vancomycin, such that 100% of the patients would have received vancomycin. Treatment success was also defined with and without adverse reactions to the antibiotic selected.[18] Readmission rates were estimated from the probability of MSSA recurrence with vancomycin and cefazolin.[14]

**Table 1:** Decision analytic model inputs.

	Base Case	Distribution	Alpha	Beta	Source
*Transition Probabilities*					
Positive Skin Test	0.14	Beta	65	400	[17]
False Positive Skin Test	0.017	Beta	2	100	
False Negative Skin Test	0.02	Beta	3	100	
Treatment Success with Cefazolin	0.927	Beta	84	5	[14]
Treatment Success with Vancomycin	0.821	Beta	198	38	
Adverse Reaction with Cefazolin	0.046	Beta	32	781	[18]
Adverse Reaction with Vancomycin	0.052	Beta	144	2881	
Readmission with Cefazolin	0.091	Beta	4	44	[14]
Readmission with Vancomycin	0.188	Beta	16	85	
***Utilities***					
Post-septic episode with no other issue	0.8	Beta	19.20	4.80	[19]
Disutility for Readmission	-0.008	Beta	99.19	12299.81	[20, 21]
Disutility for Adverse Event	-0.01	Beta	98.99	9800.01	[22, 23]
Death	0	Uniform	-	-	
***Costs***					
Penicillin Skin Test Procedure*	$300.00	Gamma	25	12	[24]
Inpatient antibiotics with allergy label	$500.00	Gamma	25	20	[25]
Inpatient antibiotics with no allergy label	$200.00	Gamma	25	8	
Outpatient antibiotics with allergy label	$53.00	Gamma	25	2.12	[15, 16]
Outpatient antibiotics with no allergy label	$38.00	Gamma	25	1.52	
Inpatient medical costs for MSSA	$7,466.00	Gamma	25	298.64	[28]
Outpatient medical costs following MSSA	$3,385.00	Gamma	25	135.40	
Adverse reaction to treatment	$7,947.00	Gamma	25	317.88	[29]

*Includes kit and labor

Abbreviations: MSSA–methicillin-sensitive staphylococcus aureus

All patients were assigned a baseline disease state QALY value for a post-septic episode.[19] In order to account for health related quality of life for patients experiencing an MSSA recurrence or adverse event to therapy, we applied a discounted QALY value estimated from previous studies in bacteremia.[20–23] Death was assigned a utility score of zero and no patients could experience a negative QALY.

Costs in each scenario were assigned based on disease state assumptions. All patients receiving PST would incur additional costs of the test itself and an approximation of labor and ancillary supplies to administer.[24] Costs of skin testing supplies are approximately $150 per patient.[24] Labor and ancillary supplies to administer may vary from institution to institution based on the site’s established protocol.[13, 24–26] For this study, the base case PST cost would be $300 per patient and increased to test model sensitivity to PST implementation cost increases. Patients who had a negative PST would experience the lower costs of antibiotic treatment estimated for non-PCN-allergy during the initial inpatient encounter and in the outpatient setting post-discharge based on previously published cost estimates for antibiotic regimens in each setting for patients with and without PCN-allergy.[15, 25, 27] Patients with a positive PST and patients not receiving PST would incur costs as a PCN-allergic patient during the inpatient stay and in the outpatient setting post-discharge estimated over 1 year.[15, 25] All patients would experience the direct medical costs for the initial MSSA bacteremia admission and post-discharge outpatient clinic costs related to the disease.[28] Disease recurrence would add the costs of a second hospitalization for MSSA bacteremia and post-discharge costs. The cost of an adverse reaction to drug treatment was estimated from the cost of anaphylaxis and applied to the initial inpatient encounter.[29, 30]

### Sensitivity analysis

Initial one-way sensitivity was conducted by varying the cost of providing the PST service to estimate a break-even point for both scenarios. A probabilistic sensitivity analysis (PSA) was conducted by assigning distributions for clinical effectiveness, costs for each health state, and utility adjustments for each health state (Table 1).[31] Input variability was based on published evidence where available. We applied a 10% standard deviation estimate across clinical effectiveness and utility parameters and a 20% standard deviation across cost estimates to account for the greater uncertainty with limited evidence for costs in MSSA bacteremia. The PSA used a Monte Carlo simulation of 1,000 repetitions of the model using the parameter distributions in Table 1.

## Results

Over a 1-year time horizon, PST services applied to all MSSA bacteremia patients reporting a PCN-allergy would result in a total cost per patient of $12,559 and 0.73 QALYs while no PST services would have a higher cost per patient of $13,219 and 0.66 QALYs per patient (Table 2). The resulting incremental savings of PST services per patient was $660 with an additional 0.07 QALY gained compared with no confirmatory testing. This resulted in a cost-effectiveness estimate of -$9,429 per QALY gained.

**Table 2:** Model results.

Treatment Strategy	QALY	Cost ($)	Incremental cost per QALY gained
Standard of Care	0.66	13,219	DOMINATED*
Penicillin Allergy Skin Test **	0.73	12,559	-

*A dominated strategy is less effective and more costly

**Assumes confirmatory testing on all patients admitted with a self-reported allergy to penicillin

Abbreviations: QALY–quality-adjusted life-year

Varying the cost of implementing PST services determined a break-even point of $959.98 where any PST cost less than this amount would actually be cost-saving and thereby dominating the no-PST decision. 95% of the incremental cost calculations in the PSA fell between [-$5,141, $3,476] and 95% of the incremental effects fell between [-0.187 QALYs, 0.319 QALYs]. Assuming a willingness-to-pay threshold of $50,000/QALY, 84% of the iterations would have determined PST to be cost-effective. At a $100,000/QALY threshold, 78% of the iterations would have determined PST was cost-effective. QALY gains were observed in 72% of the PSA iterations. In 60% of the PSA iterations, the use of PST was cost-saving when the willingness-to-pay for QALY gains was set at $0.

## Discussion

The cost-effectiveness estimate provided in the base-case model of PST services for all self-reported PCN allergic patients with MSSA bacteremia was well below generally accepted cost-effectiveness thresholds and actually estimated to be cost-saving.[32] Considering potential inpatient and outpatient health sector costs over a 1-year time horizon, PST services provide a dominant strategy in that better health outcomes are achieved for less money when compared to treatment decisions assuming the patient has a true PCN allergy. Our one-way sensitivity analysis of PST implementation costs demonstrates that the service would remain dominant up until it reaches a $960 price point. Once the total cost to purchase the supplies and provide the service exceeds $960, the cost-benefit determination would vary based on individual payer willingness-to-pay thresholds for a QALY.[32] This is an important point for institutions evaluating the decision to provide more routine PST services considering the variability of implementation strategies could influence the total cost of implementation.[13, 24–26] Interventions are generally viewed as cost-effective when they fall below thresholds such as $50,000/QALY, $100,000/QALY, or $150,000/QALY.[33] In this case, costs of the intervention may be increased substantially and may still be acceptable to payers based on QALYs gained in this short-term model.

Several studies have suggested de-labeling patients of PCN allergy through confirmatory testing may optimize antibiotic therapy by reducing the use of broad spectrum agents and overall antibiotic costs as many beta-lactams are typically less expensive agents.[13, 16, 25, 34–36] In 2015, Blumenthal et al. simulated clinical outcomes of patients with MSSA bacteremia and a self-reported PCN allergy arguing a full allergy evaluation with skin testing yields the highest rate of clinical cure and lowest MSSA recurrence.[14] This study expands on the model presented by Blumenthal and colleagues to demonstrate the potential financial impact of implementing PST services in this population. Focusing on MSSA bacteremia over all patients receiving an inpatient antibiotic reduces the variability of patients, comorbidities, and treatment options to increase the internal validity of the PST evaluation. Additional research in broader populations may be warranted to determine whether PST services should be offered to any patient presenting with a PCN allergy on admission or whether confirmatory testing should be the standard in outpatient care.

Confirming or removing a PCN allergy label while the patient is admitted helps guide the current treatment decision and has the potential to improve treatment decisions well beyond 1 year. However, we chose to model a single year to aid in payer decision-making as an insurance company may want to estimate the potential impact of paying for a new program in the current year. For patients within one health system, the medical record would reflect the PST results in perpetuity offering potential long-term implications from the one-time test. Providing a patient-friendly pocket card of the PST results may improve allergy history communication across systems but was not considered in this conservative model.

This study was also limited by its focus on the health sector costs. Future studies may consider additional costs important from the patient perspective which may include productivity losses from missed work, transportation to the hospital and clinic, and caregiver burden.[37] Additionally, this study focuses on PST implementation in an acute care setting and does not consider the use of outpatient parenteral antimicrobial therapy or nafcillin use for bacteremia. The costs of providing PST services may be less expensive in the outpatient setting or as a part of more routine care. As an outpatient service, PST could be billed separately rather than being included within a capitated inpatient payment. The costs for patient receiving vancomycin only focused on the acquisition costs of the drugs and did not account for the therapeutic drug monitoring costs frequently observed while a patient is on therapy.[25] Due to the variability of costs based on how drug monitoring practices are implemented, we relied on published cost estimates related antibiotic therapy only. Adding the costs of drug monitoring with vancomycin may increase the overall cost-effectiveness of PST services.

The probability estimates for MSSA treatment success were derived from other models that focused on effectiveness but did not consider costs.[14] Additionally, in real world settings the prescribing physician may go ahead and prescribe cefazolin over vancomycin in cases without the confirmatory testing based on the patient’s history. Accounting for history-guided treatment with cefazolin in the no-PST arm may reduce the incremental cost-effectiveness of an “all PST” versus “no PST” comparison. This may support the argument for challenging PCN-allergic patients with a cephalosporin when risk of reaction is low and saving the PST expense altogether.

As a decision-tree, patient-level factors were not included other than the PCN-allergy label itself. Future analyses may consider different methods (e.g. discrete event simulation) to test the impact of other patient characteristics. The PSA replicated the model 1,000 times with evidence-based variability in the cost and effect inputs which resulted in 16% of the iterations determining PST services were not cost effective at a $50,000/QALY threshold. Decision-makers should be mindful that the overall cost-effectiveness results of PST services in a short-term model are sensitive to the underlying assumptions.

## Conclusion

Beta-lactams are the gold-standard treatment selection for MSSA bacteremia, however, patients reporting a PCN allergy may receive sub-optimal alternative therapies such as vancomycin. The model conducted in this study identified that an inpatient PST service is cost-effective for patients with MSSA bacteremia.

## Supporting information

S1 File PST Model—Open Source PLOS ONE. (XLSX)

## Abbreviations

MSSA: methicillin-sensitive *Staphylococcus aureus*
PST: penicillin skin test
PCN: penicillin
PSA: probabilistic sensitivity analysis
ADE: adverse drug event
ABX: antibiotics
INP: inpatient
OUT: outpatient
QALY: quality-adjusted life year
ICER: incremental cost-effectiveness ratio
